# Notes and News

**Published:** 1899-01-28

**Authors:** 


### Notes and News.

(Collected and Communicated.)

HOSPITAL MEETINGS.

Italian Hospital.—The Annual General Meeting will be held on Saturday, January 28th, at 4.30 p.m., at the Italian Embassy, 20, Grosvenor Square.

After-Care Association for Poor Persons Discharged from Asylums for the Insane.—The Annual Meeting will be held at the house of Sir Samuel Wilks, Bart., Grosvenor Street, W., on Monday, February 6th, 1899, at 3 p.m., Sir Samuel Wilks in the chair.

The Royal Army Medical Corps is to have a new badge. It will consist of two branches of laurel, encircling a serpent twined round a staff, the whole surmounted by a Royal Crown, and the motto at the base runs, *In arduis fidelis.*

The crusade against tuberculosis is taking practical form in many directions. At the Liverpool Infirmary the authorities have covenanted with their milk contractor that the supply shall be obtained only from cows which have undergone the tuberculin test.

Sir William Broadbent presided at the annual meeting of the British Medical Benevolent Fund, when Dr. Samuel West reported that out of the 184 applicants 158 had been assisted. Donations and subscriptions were slightly larger than during the previous year.

The Working Men's Committee of the Grimsby Hospital have proved themselves actively interested in the institution. Their contribution this year of £270 is much in excess of that of the previous year, and they have besides provided for the painting of the children's ward.

In the Court of Queen's Bench on January 24th the appeal of Mr. H. K. Hunter, who was convicted under the Medical Act, 1858, of wilfully and falsely pretending to be, and taking and using the name and title of physician contrary to section 40 of that Act, was heard. The conviction was quashed. This was the case in which a Licentiate of the Society of Apothecaries had assumed the title of physician. It was held by the Court that such a person was not entitled so to describe himself. The conviction, however, was quashed, as the description, although false, was not wilfully false. The outcome is that an L.S.A. cannot be called a physician.

Some striking statistics in favour of inoculation for the plague are forthcoming from the town of Dharwar, in the Southern Mahratta Country. The results have been carefully collected, and it is recorded that out of 4,200 persons not inoculated 1,057 were attacked by the plague, with 827 fatal cases; whilst only 106 out of 5,963 inoculated persons were attacked, with 37 deaths.

The portable plague hospitals to be supplied in Colombo are being manufactured at the Government factory. The hospitals are to be made of iron, 24 ft. in length by 15 ft. in breadth, and 7 ft. in height. These "sheds" are so constructed as to be capable of erection in an hour's time. Kitchen and bath-room can be added to them.

The Jenner Society is issuing a large amount of literature, principally in pamphlet form, on facts relating to vaccination and small-pox. Some very useful information has been collected from old parish registers and elsewhere, which contain such appalling accounts of past ravages by the disease that it is a pity they cannot be scattered broadcast amongst the conscientious objectors, who, even in Gloucester, in spite of the awful visitation, are more numerous than in most other places.

The Milroy lectures, under the auspices of the Royal College of Physicians, will be delivered by Dr. G. Vivian Poore, on February 23rd, 28th, and March 2nd, at the College, at 5 p.m. The subject is "The Preservation and Destruction of Contagia." The Goulstonian lectures, on "The Pathology of the Thyroid Gland," will be delivered on March 7th, 9th, and 14th, by Dr. George Murray, at the same place and time; and the Lumleian lectures will be delivered by Dr. Samuel Gee, on March 16th, 21st, and 23rd, on "Diseases of the Respiratory Organs."

One more attempt to stop the erection of an infectious diseases hospital for Guildford, Godalming, and Woking has failed. The attempt on the last occasion consisted of an application to the Court of Appeal on behalf of all persons having commonable rights over Whitmore Common, to restrain the contractors for the hospital from conveying the necessary structural materials across the common. The Lord Justices decided that no injunction could be issued, and that as the contractors were liable for damages, any temporary injury to the surface of the common could be compensated for.

Liverpool has just been passing through the severest epidemic of diphtheria it has ever known. The mortality was very much lower than in previous epidemics. The Sanitary Committee voted £20, at the suggestion of the medical officer of health, Dr. Cameron, for the purchase of antitoxin for the use of patients unable to pay for it themselves. At a recent meeting of the Leeds and West Riding Medico-Chirurgical Society a very large number of members attended, and all speakers who joined in the discussion testified to the value of the serum treatment in diphtheria. As we pointed out last week, the use of antitoxin by club doctors is almost prohibited by its price, and some prompt public assistance, as in the case of Liverpool, should always be forthcoming.

